# Sturge Weber syndrome in a multinational pediatric cohort: a systematic analysis of different types

**DOI:** 10.1186/s13023-025-03769-2

**Published:** 2025-07-02

**Authors:** Sigrid Disse, Georgia Ramantani, Hanna Küpper, Annette Bock, Georg-Christoph Korenke, Birgit Weidner, Martin Preisel, Regina Trollmann, Adelheid Wiemer-Kruel, Sven Wellmann, Knut Brockmann, Simone Schroeder, Sascha Meyer

**Affiliations:** 1 University Children’s Hospital Regensburg (KUNO) – Hospital St. Hedwig of the Order of St. John, University of Regensburg, Linik St. Hedwig, Steinmetzstraße 1-3, 93049 Regensburg, Germany; 2 Department of Neuropaediatrics, University Children’s Hospital Zürich, Zürich, Switzerland; 3 University Children’s Hospital Tübingen, Tübingen, Germany; 4 University Hospital Essen, Essen, Germany; 5 Department of Neuropaediatrics, University Children’s Hospital, Klinikum Oldenburg, Oldenburg, Germany; 6 Paediatric Practice, Cottbus, Germany; 7 University Hospital Salzburg, Salzburg, Austria; 8 Paediatric Neurology, University Children’s Hospital, Erlangen, Germany; 9 Paediatric Epilepsy Center Kehl-Kork, Kork, Germany; 10 University Medical Center Göttingen and German Center for Child and Adolescent Health (DZKJ), Göttingen, Germany; 11 Franz-Lust Klinik Für Kinder Und Jugendliche, Karlsruhe, Germany

**Keywords:** Sturge-Weber Syndrome, Phacomatosis, Observational study, Roach classification, Facial portwine birthmark

## Abstract

**Background:**

Sturge-Weber Syndrome (SWS) is characterized by leptomeningeal capillary malformation (CM), glaucoma, and facial vascular birthmark. The Roach Scale differentiates between cases with facial birthmark (Roach Type I) versus isolated brain involvement (Type III). Most previous studies have focussed on classic SWS Type I, but Type III cases were mostly described in case reports. We systematically compare cases with and without facial birthmark, with a focus on epilepsy variables, cerebral involvement and overall outcome.

**Methods:**

Using a cross-sectional observational study conducted through a well-established child neurologists’ network, we recruited pediatric patients with clinically diagnosed SWS from Germany, Switzerland, and Austria. The patients’ guardians and attending child neurologists filled in detailed questionnaires. All patients were classified according to the Roach classification by both attending child neurologists and the study team.

**Results:**

Our study identified 47 pediatric SWS patients (participation rate 43.2%). 35 cases (74.5%) fulfilled the criteria for classic SWS; six cases (12.8%) showed no skin involvement, the remaining cases were overlap/atypical phacomatoses with leptomeningeal and facial CM. Male/female ratio was 1.14, age ranged between 115 days and 17 years. Cases without facial birthmark were older at diagnosis (p = 0.005), and none showed ophthalmologic involvement. Comparison of age at first seizure did not reach significance after adjustment (p = 0.026). There was no significant difference between SWS types with regard to seizure types or frequency number of antiseizure medication (ASM), epilepsy surgery, cerebral involvement, SWS neuroscores. Multivariable analysis showed that, seizure frequency was independent of SWS type and epilepsy surgery, but was positively associated with the number of ASM required for seizure control (p = 0.0056). 50% of operated patients were seizure-free at study inclusion.

**Conclusions:**

Type I and Type III SWS cases showed comparable profiles with regard to different epilepsy features, SWS neuroscores and number of used ASM. Type III patients were older at diagnosis and showed no ophthalmologic involvement, compatible with a milder SWS phenotype. Only few patients were evaluated for surgery, despite uncontrolled, structural epilepsy. Larger cohorts are needed to reevaluate the effectiveness of surgical therapies in different SWS types.

**Supplementary Information:**

The online version contains supplementary material available at 10.1186/s13023-025-03769-2.

## Introduction

Sturge-Weber Syndrome (SWS) belongs to a heterogenous group of rare diseases widely known as neurocutaneous diseases. These conditions show both manifestations of the skin and of the brains as a shared feature. SWS may additionally include eye involvement and usually comprises at least two of these three manifestations. Clinical symptoms in SWS vary widely between affected individuals and this is partially reflected in the Roach classification for SWS [1], which dates back to 1992: Type I SWS, the “classic” type of SWS, shows “facial and leptomeningeal angiomas, may have glaucoma [1]”. Type II SWS is defined by “facial angioma without evidence of intracranial disease; may have glaucoma [1]”. In contrast, Type III lacks any skin and eye involvement and is characterized by “isolated leptomeningeal-brain angioma [and] usually, no glaucoma [1]”. Today, the most recent ISSVA (International Society for the Study of Vascular Anomalies) classification for vascular anomalies, only recently updated in 2025, classifies SWS as a “Slow-Flow, Syndromic Port wine capillary malformation” (CM) [2]. The Roach Scale [1], does not stratify risk for CNS involvement depending on facial CM characteristics, but as the Roach classification is still widely known and as it shows the clinical aspects of SWS required for our current research question, we have chosen it for the present study.

While numerous case reports depict the clinical presentations and imaging studies in patients diagnosed as SWS Roach Type III/SWS-“without facial nevus” (e.g. [3–5]), systematic clinical data on different types of SWS are scarce. In most published SWS cohorts, the proportion of SWS cases with isolated brain involvement has been stable over the past years, ranging around 4–14% [6–9], irrespective of nationality/ethnicity.

Numerous previous clinical studies and reviews have investigated skin involvement in SWS thoroughly [11–14]. In 2025, El Hachem et al. presented multidisciplinary multicenter consensus recommendations obtained by a Delphi process, including dermatological recommendations on facial localisations which should raise suspicion towards SWS [15]: As described by Waelchli et al. [12], a facial CM involving any part of the “forehead” region, “delineated at the lateral and inferior margins by a line joining the outer canthus of the eye to the top of the ear, and including the upper eyelid”, is associated with the highest risk for SWS. Of particular importance, Waelchli et al. also showed that this forehead region corresponds to distinct embryologic facial structures and their developing vasculature [12]. A classic hemifacial and a distinct median facial pattern were also shown to be high-risk localisations by another group [13], but they largely involve the previously described forehead region. Due to these recent advances showing that SWS pathogenesis is closely linked to embryologic development, facial cutaneous involvement in SWS is no longer described using trigeminal nerve territory. Whether the laterally or the median frontally located facial portwine birthmarks (FPBs) are associated to higher SWS risk, currently still is under investigation [11]. Furthermore, in patients *with known SWS*, the size of the facial CM was shown to correlate with clinical and radiologic disease severity [14]. Following consensus statements by 13 U.S. experts on SWS, routine imaging in asymptomatic infants with high-risk FPB is no longer recommended, but may be considered in patients with particularly high risk for seizures, e.g. in suspected bilateral SWS [16]. Consistent with that, the most recent multidisciplinary consensus recommendations state that, to ascertain the diagnosis SWS, contrast-enhanced MRI remains the “gold standard” [15].

To the best of our knowledge, Powell et al. [17] (published in 2021) were the first to systematically compare facial portwine birthmark positive and FPB negative cases with SWS in a large (n = 140) retrospective national U.K. cohort. They hypothesized similar outcomes in both SWS types due to the common somatic mutation. However, as a main finding, FPB-negative cases in this study showed less extensive leptomeningeal CM, better language and cognitive outcomes, and absence of glaucoma [17]. The numbers of episodes with status epilepticus and seizure clusters were comparable between the two groups despite significantly earlier status epilepticus onset in FPB positive cases. The finding was explained by the authors through the shared, disease-causing GNAQ mutation [18–20]. Pathogenesis of SWS is also reviewed by El Hachem et al. [15]. Since the forementioned, somatic activating mutation in guanine nucleotide-binding protein alpha-q subunit encoded by GNAQ gene on chromosome 9q21 was first described to be causative by Shirley et al. [18] as the—to date most common—molecular cause of SWS in 2013, further research has identified two other, more rarely occurring mutations: Firstly, in guanine nucleotide binding protein alpha-11 subunit encoded by GNA11 gene located on chromosome 19p13 [21], and secondly, in the beta chain of the same heterotrimeric G-protein encoded by GNB2 gene located on chromosome 7q22.1 [22]. Ultimately, discovery of new mutations and description of the respective phenotypes [21, 25] has contributed to a deeper understanding of the pathophysiology, the broad clinical spectrum and of genotype–phenotype correlations in SWS. As outlined by Wu et al., detection of the causative GNAQ mutation in affected tissue, such as episcleric tissue from SWS patients with secondary glaucoma [26], may also serve as diagnostic and/or therapeutic targets in the future. Today, genetic testing, e.g. from skin biopsies, is not part of routine diagnostics in SWS [27], but it has proven to be beneficial in atypical cases [28].

Yet, the biology of the FPB negative cases remains to be fully understood. In brain specimens obtained during epilepsy surgery, digital droplet PCR detected the same GNAQ R183Q mutation with low mutation frequency in 4/4 FPB negative-SWS patients—which are often referred to as “forme fruste” of the condition [20]. In summary, current state of knowledge on the role of different clinical subtypes of SWS, i.e. with and without FPB, is scarce. We used data from our comprehensive database built in our previous clinical study [29] to expand current understanding of these two main subtypes of SWS and to support patient/parent counselling. We hypothesize that similar types of symptoms in FPB-negative and FPB positive cases may occur, but with an overall milder phenotype in the FPB-negative subcohort. This study retrospectively compares different types of SWS in our previously described multinational cohort of 47 pediatric SWS patients [29] with regard to epidemiologic features, different epilepsy variables, neurocognitive function as assessed by previously described SWS neuroscores [30] and with regard to antiseizure medication (ASM) and epilepsy surgery data.

## Materials and methods

We conducted a multinational, cross-sectional study in Germany, Switzerland, and Austria to recruit pediatric SWS patients. For the study, we used an established network of child neurologists (“ESNEK,” i.e. in German “Erhebung Seltener Neurologischer Erkrankungen im Kindesalter”; engl. “Registry of Rare Neurological Disorders in Childhood”) [31]. We have given detailed descriptions of the study method and procedures in our previous studies [20, 22]. We registered the study in the German Clinical Trials Registry (ID: DRKS 00013551, UTN U1111-1206-9923). The study protocol was approved by the responsible Institutional Review Board in Saarbrücken, University of Saarland, Germany (ID 209/17, October 2017). Altogether, 49 child neurologists notified us of 111 SWS patients in their attendance. Via the German SWS support group or by personal contacts between patients, 10 patients self-recruited. Their diagnoses were verified by our study team. Altogether, 47 patients were included in our study. The patients’ legal guardians gave informed consent in 44 cases. Three cases were sent anonymously by their attending child neurologists. As all data were stored anonymously, the above-named responsible Institutional Review Board in Saarbrucken, Germany waived informed consent in these cases (02/2023). All tables and texts which present single patients in detail (Appendix, Table 5, see below), include only patients whose parents/caregivers gave informed consent. To conclude, all obtained data were used in accordance with the General Data Protection Regulation of the European Union (“Datenschutz-Grundverordnung”) and the responsible Institutional Review Board.

This study included all patients aged < 18 years at the time of study inclusion with clinically diagnosed SWS who currently live in Germany, Switzerland, or Austria. Following internationally recognized SWS criteria by Roach et al. [1], we enrolled patients with SWS types I-III. In order to present the broad clinical spectrum of SWS, we also included cases which showed an additional overlap with other phacomatoses or presence of systemic CM. Detailed clinical data of these, designated “atypical” cases are presented in detail in the Appendix (Table 5). In this context, it should be emphasized that all “atypical” patients display full characteristics of SWS including leptomeningeal and skin CM, as well as glaucoma.

All patients were enrolled between January 2018 and December 2018; a few cases reported late until June 2019. For all cases, we verified the collected data through the attending child neurologists (Table 1).

**Table 1:** Patient characteristics for 47 SWS patients from Germany, Switzerland and Austria by Roach Types I–III and atypical cases

Patient characteristics (n = 47)	N (%)—all types	SWS Roach Type I (n = 35), %	SWS Roach Type III (n = 6), %	Atypical cases (n = 6), %
Sex				
Male	25 (53.2)	18 (51.4)	3 (50.0)	4 (66.7)
Female	22 (46.8)	17 (48.6)	3 (50.0)	2 (33.3)
Current age in years, median (IQR); range	4.2 years (1.9–9.0); 4 months–17.5 years	4.2 years (1.9–10.6); 4 months–17.5 years	5.5 years (5.0–6.5); 3–11.6 years	2.1 years (1.4–3.2); 4 months–4.3 years
Age at first diagnosis				
In months, median (IQR); range	5.0 months (0.2–9.0); 0–24 months	4.5 months (0.2–8.3); 0–20 months	18.5 months (10.5–20.5); 6–24 months	1.5 months (0–3.8); 0–11 months
Ethnicity (not reported = 14)				
Caucasian	30 (90.9)*	23 (65.7)	4 (66.7)	3 (50)
Asian/Arab	2 (6.1)*	2 (5.7)	0	0
African (mother)	1 (3.0)*	0*	1 (16.7)*	0*
Country				
Germany	39 (83.0)	29 (82.9)	5 (83.3)	5 (83.3)
Austria	4 (8.5)	3 (8.6)	0	1 (16.7)
Switzerland	4 (8.5)	3 (8.6)	1 (16.7)	0

There were no cases classified as Roach Type II. Data on ethnicity are incomplete, resulting in missing values and column sums < 100%

We used two modified versions of our previously described questionnaires [32] to build up a comprehensive database. One questionnaire each was adapted for caregivers, and one for child neurologists. The caregiver’s questionnaire comprised questions on patients’ demographics, family history, birth and prenatal history, ethnicity, current symptoms, including organ involvement, hearing, feeding, language skills, neurocognitive development, and use of health services. The child neurologists’ questionnaire included questions on SWS-specific organ involvement—under consideration of recent findings [14], family history, birth and prenatal history, current symptoms including components of SWS clinical severity scores [30], further organ involvement, diagnostic procedures incl. genetics, therapies and therapeutic success, including the use of ASM and epilepsy surgery. Clinical severity scores [14, 30] comprise the severity of visual field defect, hemiparesis, seizure frequency and cognitive function, were calculated from the child neurologists’ questionnaires. As paper questionnaires allowed users to skip questions, we formally handled some missing data as normal values in the section on SWS clinical severity scores, if this section was otherwise complete and the given values medically plausible. We used a previously [33] demonstrated cut-off value to distinguish between intellectually impaired patients (score ≥ 4) and non-impaired patients (score < 4; sensitivity 75%, specificity 65%).

For some questionnaire items, we acknowledge missing data as follows: epileptic seizures during lifetime (n = 1), paresis (n = 3), need of visual aid (n = 5), laterality of FPB (n = 3), data on neuroscore incomplete and thus not included (n = 12). Data on cerebral atrophy in cerebral MRI not explicitly documented (n = 5), on EEG diagnostics (n = 1), and some questionnaires lacked a formal diagnosis of developmental status/delay (n = 6).

### Statistical methods

Data analysis was exploratory. Systematic comparisons were conducted between Roach Type I and Type III patients for We used RStudio (2024.9.0.375’), R version 4.4.1 (2024-06-14 ucrt) for data analysis [34]. For normally distributed numerical covariates, we each indicated mean and standard deviation. If the assumptions were not fulfilled, we used median/interquartile ranges. For categorical variables, absolute and relative frequencies are given. We used Exact Fisher’s test or Chi Square test-depending on expected cell frequencies—to evaluate the independence of categorical variables.

As a multivariable analysis, logistic regression evaluated the association between seizure frequency as a dependent (dichotomized) variable, and SWS type, epilepsy surgery and the number of ASM as predictor variables. We chose the following two groups for dichotomization of seizure frequency, i.e. group 1 (= controlled epilepsy/seizures): patient never had seizures OR one/more seizures but now seizure-free; group 2 (= uncontrolled epilepsy/seizures): breakthrough seizures, monthly seizures, OR weekly seizures or more. Analysis of Generalized Variance Inflation factors showed no relevant multicollinearity of the model.

The threshold for statistical significance was set at p < 0.05. To adjust for multiple testing, we performed Benjamini Hochberg procedure with a false discovery rate of 0.2 (as in a previous study on SWS [17]).

Due to the very small number of operated patients, we conducted a retrospective statistical power calculation for the detection of a significant difference in seizure frequency between surgical and non-surgical patients. The calculation was approximative, based on a Welch’s t-test for unequal variances with the following parameters: the observed effect size Cohen’s d = 0.12, the observed unequal sample sizes of n1 = 4 and n2 = 42, and a significance level of alpha = 0.05. It showed that, under the conservative assumptions of Welch’s t-test, retrospective power for this question was insufficient in this cohort, i.e. only 55.3%.

## Results

### Overview of the study cohort

Following 111 notifications of non-related pediatric patients with clinically diagnosed SWS, 47 patients fulfilled the inclusion criteria, consented to participate in our survey and completed our questionnaires (response rate 43.2%). Twenty-five patients were male, 22 patients were female (ratio m/f = 1.13). Median age was 4.0 years (1.0–8.5 years), the age span ranged from 115 days to 17 years. 35 patients showed facial birthmark () and leptomeningeal capillary malformation (CM), and were classified as Roach Type I. Six patients fulfilled criteria for Type III and the remaining 6 patients were overlap/atypical phacomatoses. We systematically compared Roach Type I (FPB positive) and Type III (FPB negative) cases; the patients with overlap/atypical phacomatoses are presented additionally (see Tables 2, 3, 4).

**Table 2:** Comparison of clinical characteristics of different SWS types in 47 pediatric patients showing median and interquartile range for numerical variables, and absolute and relative frequencies for categorical variables

Patient characteristics	N (%)—all types (n = 47)	SWS Roach Type I (n = 35), %	SWS Roach Type III (n = 6), %	Atypical cases (n = 6), %	Comparison Roach Type I versus Roach Type III
Histories					
Maternal age at birth (years)	30.5 (27.0–33.0)	30.0 (27.0–33.0)	32.5 (29.0–33.8)	33.5 (30.3–37.5)	p = 0.32
Diagnostics					**p = 0.005**
Age at diagnosis (months)	5.0 (0.2–9.0)	4.5 (0.2–8.3)	18.5 (10.5–20.5)	1.5 (0–3.8)	
Medical discipline that made the diagnosis (order of relative frequencies)	1. Children’s hospital 2. Child neurologist 3. Birth clinic	1. Children’s hospital 2. Birth clinic 3. Child neurologist	1. Children’s hospital 2. Child neurologist 3. radiologist	1. Children’s hospital 2. Birth clinic/child neurologist/other*	
Epilepsy/seizures					
Age at first seizure [months]	6.5	6.0 (4.0–9-0)	13.0 (13.0–14.0)	7.3 (2.3–11.9)	**p = 0.026**
Current seizure frequency					p = 0.091
Never had a seizure	4 (8.5)	3 (8.6)	0	1 (16.7)	
≥ 1 prior seizure, now seizure-free	19 (40.4)	17 (48.6)	2 (33.3)	0	
Breakthrough seizures	4 (6.5)	1 (2.9)	2 (33.3)	1 (16.7)	
Monthly seizures	6 (12.8)	5 (14.3)	0	1 (16.7)	
Weekly seizures or more	6 (12.8)	5 (14.3)	0	1 (16.7)	
Unknown	8 (17.0)	4 (11.4)	2 (33.3)	2 (33.3)	p = 0.692
Types of seizures					
Focal	21 (44.7)	15 (42.9)	5 (83.3)	1 (16.7)	
Generalized	5 (10.6)	5 (14.3)	0	0	
Focal and generalized	13 (27.7)	9 (25.7)	1 (16.7)	3 (50.0)	
Antiseizure medication					
Number of ASM (median, IQR)	2.0 (1.0–2.0)	2.0 (1.0–2.0)	2.0 (1.3–2.0)	2.0 (1.3–2.8)	p = 0.924
Use of aspirin	21 (44.7)	15 (42.9)	3 (50.0)	3 (50.0)	not eval
Epilepsy surgery					
Surgery performed**	4 (8.5)	3 (50.0)	1 (16.7)		p = 0.65
Type of surgery	1: hemispherectomy 1: hemispherotomy 2: cortical excision	1: hemispherectomy 1: hemispherotomy 1: cortical excision	1: cortical excision		

Sums < 100% are due to missing values and rounding

*Other: caregiver reported common diagnosis by internal specialist and dermatologist

**2 further patients were evaluated for epilepsy surgery, 1 further patient was planned for surgery at study inclusion

**Table 3:** Comparison of clinical characteristics of different SWS types in 47 pediatric patients

Patient characteristics	N (%)—all types (n = 47)	SWS Roach Type I (n = 35), %	SWS Roach Type III (n = 6), %	Atypical cases (n = 6), %	Comparison Roach Type I versus Roach Type III
Cerebral involvement					p = 1.0
Cerebral atrophy	35 (74.5)	25 (71.4)	4(66.7)	6 (100)	
Cerebral calcifications	18 (38.3)	16 (45.7)	1 (16.7)	1 (16.7)	
Stroke-like episodes	8 (17.0)	3 (8.6)	2 (33.3)	3 (50.0)	
Migraine	7 (14.9)	7 (20)	0	0	
Neurocognitive status/development					
SWS neuroscore	6.0 (3.0–9.0)	6.0 (4.0–9.0)	3.0 (2.8–3.8)	7.5 (4.5–9.3)	p = 0.164
Cognitive impairment					p = 0.158
None	11 (23.4)	6 (17.1)	4 (66.7)	1 (16.7)	
Slight	12 (25.5)	8 (22.9)	2 (5.7)	2 (33.3)	
Moderate	6 (12.8)	4 (11.4)	0	2 (33.3)	
Severe	12 (25.5)	12 (34.3)	0	0	
Paresis*					p = 0.297
0: none	16 (34.0)	12 (34.3)	3 (50.0)	1 (16.7)	
1: mild, body posture affected	6 (12.8)	4 (11.4)	1 (16.7)	1 (16.7)	
2: only fine motricity	5 (10.6)	3 (8.6)	2 (33.3)	0	
3: significant, fine and gross motricity	11 (23.4)	10 (28.6)	0	1 (16.7)	
4: severe, fine and gross motricity	6 (12.8)	5 (14.3)	0	1 (16.7)	
Language development					p = 0.160
Normal	17 (36.2)	11 (31.4)	5 (83.3)	1 (16.7)	
Speech difficult to understand	1 (2.1)	1 (2.9)	0	0	
Autistic	1 (2.1)	1 (2.9)	0	0	
No speech	4 (8.5)	4 (11.4)	0	0	
Too young	3 (6.4)	2 (5.7)	0	1 (16.7)	
Complex, but mild impairment	11 (23.4)	9 (25.7)	0	2 (33.3)	
Complex, severe impairment	1 (2.1)	0	1 (16.7)	0	

Unless otherwise stated, median and interquartile range (in brackets) are given

*For 1 patient each, child neurologists reported paresis scores of 1.5 and 2.5, here classified/rounded to 2.0 and 3.0. Sums < 100% are due to missing values and rounding

**Table 4:** Comparison of clinical characteristics of different SWS types in 47 pediatric patients

Patient characteristics	N (%)—all types (n = 47)	SWS Roach Type I (n = 35), %	SWS Roach Type III (n = 6), %	Atypical cases (n = 6), %	Comparison Roach Type I versus Roach Type III
Ophthalmologic involvement*					
Congenital glaucoma	14 (30.0)	9 (25.7)	0	5 (83.3)	p = 0.079
Glaucoma in further course	9 (19.1)	7 (20.0)	0	2 (33.3)	p = 0.669
Visual field loss	15 (31.9)	12 (34.3)	2 (33.3)	1(16.7)	
Blindness	2 (4.3)	2 (5.7)	0	0	
Retinal or corneal pathology	2 (4.3)	2 (5.7)	0	0	
Therapy for glaucoma					
Surgery	1 (2.1)	1 (2.9)	0	0	
Drugs	7 (14.9)	5 (14.3)	0	2 (33.3)	
Combined surgery and drugs	12 (25.5)	8 (22.9)	0	4 (66.7)	
Other	1 (2.1)	1 (2.9)	0	0	
Use of visual aid	16 (34.0)	11 (31.4)	1 (16.7)	4 (66.7)	
Supportive measures**					p = 1.0
Physical therapy	34 (72.3)	26 (74.3)	3 (50.0)	5 (83.3)	
Speech therapy	19 (40.4)	13 (37.1)	3 (50.0)	3 (50.0)	
Manual therapy	25 (53.2)	19 (54.3)	4 (66.7)	2 (33.3)	

Unless otherwise stated, median and interquartile range (in brackets) are given

*Visual field loss is usually of central origin, but perimetry is usually performed by an ophthalmologist

**Combinations possible

### Epilepsy in different types of SWS

Most patients of the cohort (91.5%) had at least one seizure until study inclusion (43/47). In Roach Type III patients, two out of six were currently seizure-free after one or multiple seizures in the past, and further another two suffered from breakthrough seizures (2 NAs). In Roach Type I patients, 48.6% were currently seizure-free after one or multiple seizures in the past (17/35), and 2.9% suffered from breakthrough seizures (1/35). None of the Type III patients reported monthly or weekly seizures. However, in 14,3% (5/35) of Roach Type I patients, seizures occurred at least once per month, and in further 14,3% (5/35) seizures occurred at least once per week.

Roach Type I patients were younger at first seizure (median age 6.0 months) than Type III patients (median age 13 months). The difference did not remain significant after adjustment (p = 0.026).

About seizure type, most Roach Type III patients reported mostly focal seizures (83.3%, 5/6), rarely both generalized and focal seizures. In Roach Type I patients, focal seizures were the most frequent type of seizures as well (42.9%, 15/35), and comparison of seizure type did not reach statistical significance (p = 0.692). Further details are presented in Tables 2, 3, 4.

### Cerebral involvement, SWS neuroscores and cognitive impairment in different types of SWS

Cerebral atrophy on MRI was common, irrespective of SWS Type (74.5% of the whole cohort), with no relevant difference between Roach Types I and III. Notably, brain atrophy was documented in all atypical cases (100%, 6/6). Calcifications occurred more frequently in classic Type I (45.7%) than in Type III (16.7%). Migraine was only reported in Type I cases (20%).

Median SWS neuroscore was lower in Type III patients (3.0, IQR 2.8–3.8) than in Type I (median 6.0, IQR 4.0–9.0) and in the atypical cases (median 7.5, IQR 4.5–9.3).

Only approximately one quarter of the cohort showed no cognitive impairment at time of study inclusion (23.4%), a finding which was more frequent among Type III cases (66.7% of Type III cases) than among Type I cases (17.1% of Type I cases). Comparison of overall cognitive impairment remained statistically not significant (p = 0.158).

### Ophthalmologic involvement in different types of SWS

Congenital glaucoma was reported in 30% of the whole cohort (14/47). None of Type III cases was affected. Another 19% of the cohort developed non-congenital glaucoma after birth(9/47), again, only Type I and atypical cases, but no Type III cases. Notably, age of onset in these cases of non-congenital glaucoma varied widely, ranging from eight weeks to twelve years. Non-congenital glaucoma cases included two atypical SWS cases which first manifested at the age of four weeks and 14 months, respectively.

In most cases of glaucoma (irrespective of age at onset), therapy included combined surgery and drugs (25.5% of the cohort), sometimes drugs only (14.9%).

The proportion of patients with a visual field deficit was approx. one third of the cohort, with no relevant difference between Type I and Type III patients. Two patients’ visual acuity was reported to be equivalent to blindness; both were classic Type I patients. Further details on ophthalmologic involvement are displayed in Table 4.

### Antiseizure medication (ASM), use of aspirin and epilepsy surgery in different types of SWS

In the whole cohort, median number of ASM used was 2.0 (IQR 1.0–2.0). There was no relevant difference between the different SWS types (p = 0.924). Aspirin was administered irrespective of SWS type.

Four patients received epilepsy surgery prior to our survey, i.e. two had cortical excision, one hemispherotomy, one hemispherectomy. Surgery was performed both in Type I cases (n = 3) and in one Type III case. Details on the subcohort of patients with received surgery are given below.

### Post-hoc subcohort analysis: Cases with epilepsy surgery

All four cases which received epilepsy surgery were female. 75% of operated patients were SWS Type I (n = 3), and one was Type III. Age ranged from 4 to 17 years, with a median age of 13.5 years. All patients displayed cerebral CM, cerebral atrophy, and two had cerebral calcifications. Another two patients suffered from stroke-like episodes, both with significant sequelae. Severity of paresis in these patients ranged from grade 3–4.^1^ SWS neuroscores in the operated subcohort ranged from 5–11. Two of the operated patients also suffered from congenital glaucoma. The number of currently required ASM ranged from 0–3, and none of the patients received additional aspirin. After surgery, at least two cases were “seizure-free” at the time of study conduct, one patient still reported “weekly seizures” (NA = 1 for this item, in a normally developed Type III patient without paresis). The number of used ASM in operated patients were 0, 1, 2 and 3, respectively. The median number of ASM in nonsurgical patients was 2.0 (IQR 1.0–2.0, range 0–4), but the very small number of operated patients precludes statistical tests for comparisons. Figure 1 (online-content only) illustrates the number of ASM in surgical and non-surgical patients.

### Multivariable model of seizure frequency as a function of SWS type

Our multivariable model included SWS type, current number of ASM and epilepsy surgery as predictors of seizure frequency. Binary logistic regression showed a significant, positive association between seizure frequency and the number of administered ASM (p = 0.0056), with a regression coefficient of 1.33 (i.e. OR 3.77, 95% CI 1.64; 11.22). SWS type and epilepsy surgery each were not associated with seizure frequency.

## Discussion/conclusions

To the best of our knowledge, our cohort is the first multinational cohort to systematically investigate different SWS types. Comparing Type I cases (classic form) and Type III cases (no skin involvement), we present detailed clinical data on epilepsy including ASM, aspirin use and surgery, cerebral and ophthalmologic involvement, and neurocognitive outcomes.

### Key epidemiologic data of our cohort

Our pediatric cohort is composed of 47 pediatric SWS cases including 87.2% Type I (FPB positive) cases and 12.8% Type III (FPB negative) cases, and is thus comparable to the U.K. cohort by Powell et al. (85.7% FPB positive cases) [17] and to published U.S. SWS cohorts [6, 8, 9] regarding the proportion of FPB positive/negative cases. We have described other key demographic and epidemiologic data of the cohort in our previous publication [29].

### Epilepsy in different SWS types

Age at first seizure in our whole cohort (6.5 months) is also comparable to the literature^2^ [9, 17]. In concordance with these findings, Smegal et al. [6] reported a seizure onset ≤ 12 months in 196 out of 268 patients (73.1%)—in one of the to date largest cohorts. Most published SWS cohorts did not investigate epilepsy in different SWS types [6, 8]. A statistically non-significant, yet numerically later seizure onset in Type III/FPB negative patients was recorded in our own cohort, i.e. 13 months. Likewise, in the UK cohort by Powell et al. [17] (9 months) median age at onset of status epilepticus was significantly later in FPB negative patients, but the number of episodes with status epilepticus was comparable. Day et al. [9] also found a later seizure onset in FPB negative patients (17 months) versus patients with unilateral PWB (10 months) or bilateral PWB (5 months). In the literature, there is a strong agreement that early seizure onset is associated with a strong negative impact on neurocognitive development [9, 17, 35, 36]. Hence, delayed seizure onset is SWS Type III may contribute to a better intellectual function in these patients (see below).

Regarding seizure type, Our Type III cases reported nearly only “focal seizures”; “focal and generalized seizures” were rare, however, our questionnaire did not assess “focal seizures with secondary generalization”. Classic SWS Type I in our cohort also reported a predominance of focal seizures (42%), but generalized or mixed types of seizures were common in classic Type I as well. Due to our small sample size of Type III cases, statistical analysis of seizure types did not achieve statistical significance.

In the literature, many case reports, series and studies depict a predominance of focal or focal onset seizures^3^ in FPB negative patients [37] and in SWS in general [7, 36, 38, 39]. In some studies, the types of seizures are not explicitly stated [6]. The UK cohort by Powell et al. [17] specified that *first seizure semiology* was focal motor in 16 out of 20 FPB negative cases (80%), as compared to 69/120 (58%) FPB positive cases. We did not assess the frequency of seizure clusters, but this is another common type of seizures in SWS [36] that probably occurs slightly more frequent in classic SWS [17]. A predominance of focal seizures in Type III cases can be explained very well through the—as reported by Powell et al.—fewer affected cerebral lobes by the leptomeningeal capillary cerebral malformation as compared to classic SWS [17].

Seizure frequency (as a binary variable—i.e. controlled versus uncontrolled seizures) was independent of SWS type in our multivariable analysis. In our whole cohort, only 48.5% were seizure-free at study inclusion; further 32% suffered from seizures with different frequencies (for details, please see Table 2). As expected, our model showed that the odds for an additional ASM were more than threefold higher in uncontrolled versus controlled seizures (odds ratio = 3.7). In the Powell cohort, the number of seizures also did not differ between FPB positive and FPB negative patients [17].

### Neurocognitive outcomes in different SWS types

SWS is an often progressive [40] neurovascular disorder which puts neurocognitive development of affected patients at risk. In our study, two thirds of patients showed various degrees of intellectual impairment (64%), and among these, severe impairment was frequent (43%). However, two thirds of our Type III patients showed normal cognitive function (66.7%), and none of Type III patients showed severely or moderately impaired cognitive function. The findings agree well with the results from the UK cohort by Powell et al. (n = 140) [17]: 25% of FPB-positive cases were not impaired (in our own cohort: 17.1% of Type I cases not impaired), and 50% of FP—negative cases were not impaired (in our own cohort: 66.7% of Type III not impaired). Intellectual status was not addressed in some of the other cohorts [6]. However, one of the so far largest published SWS cohorts by Day et al. with 277 pediatric (85.6%) and adult participants—recruited from 7 U.S. sites—reported a far smaller proportion of patients with “intellectual disability” (14.8%), and 41.9% with a “learning disorder”—despite a relatively high proportion of patients with bilateral FPB (35.7%) and a similar proportion of Type III patients. Even so, the authors estimated their results to be “likely skewed toward the more severely involved subjects” due to patient recruitment from tertiary centres. The higher rate of impaired patients in our and the above-cited studies is most likely due to the large sample size in the Day cohort [9]. Additionally, our patients´ younger age, possibly points towards a more severe involvement and thus, a selection of more severely affected cases in our study (see below) might contribute. Click or tap here to enter text..

Analysis of 11 published cases with SWS Type III reported on as single case reports or as small series shows that their intellectual status was mostly within normal limits [3–5, 41–44]; only very few cases depicted intellectual impairment [45] or progressive deterioration [46]. This may be explained by the fact that, on average—as shown by Powell et al. [17]—Type III patients show significantly reduced brain involvement as compared to classic Type I patients, i.e. fewer lobes with CM, which is always unilateral. On a molecular basis, an intact intellectual function and less extensive leptomeningeal CM are compatible with the smaller mutant allele frequencies of the causative GNAQ gene mutation found in SWS Type III cases (0.42–7.1%) [20] as compared to the allele mutation frequency described in classic Type I cases (1–18.1%) [18]—presumably, due to a later occurrence of the shared somatic mutation.

### Diagnosis in SWS Type III

In our cohort, all Type III cases were diagnosed within the first two years of life. However, analysis of published case reports on Type III cases shows numerous late manifestations [5, 41, 44] and/or delayed diagnoses [4, 41, 44, 45], often with many years between first symptoms and final diagnosis—up to 26 years [41]: One patient showed first symptoms at the age of 6 years (with status migrainosus; initially suspected for encephalitis [4]), and was diagnosed with SWS Type III three years later. Another patient became symptomatic at the age of 10 years with headaches and visual aura and was finally diagnosed with SWS Type III at the age of 36 years [41]. Finally, a 62-year old man was diagnosed with SWS Type III after a first generalized seizure and typical MRI findings; his first manifestation three years before had been misclassified as a culture-negative “focal leptomeningitis” [47]. To conclude, diagnosis in SWS Type III can be particularly challenging and requires regular counselling of medical staff regarding clinical signs and pitfalls in cerebral imaging. Beyond that, a revision of current guidelines could potentially accelerate the diagnosis of SWS Type III patients. As outlined by others, “currently, no guidelines^4^ recommend contrast-enhanced MRI in the evaluation of focal seizures or stroke-like episodes” and it is “important to keep a high index of suspicion in cases presenting with focal epilepsy with no detectable foci in plain MRI or stroke-like episodes with normal MRI.” [49] The same may hold true for patients with migraine—a potential first symptom of SWS Type III [50]—and prolonged focal symptoms.

### Aspirin therapy and epilepsy surgery in different SWS types

In our cohort, aspirin was administered equally frequent to patients of both SWS types. This was also recorded in the U.K. cohort by Powell et al. [17]. The finding can be explained by similar occurrence of stroke-like episodes.

Epilepsy surgery included SWS patients of both types (four patients). Both surgical patients which remained seizure-free after surgery (i.e. 50% of the operated patients) were Type I SWS patients (no information available on outcomes of the operated Type III patient), and one had received hemispherotomy, the other a cortical excision. Hemispherectomies were the most frequently used surgical technique in the Powell cohort [17]. A recently published study by Ramantani et al. including 36 patients with SWS found no difference in outcomes with regard to surgical technique in pediatric hemispherotomy [51].

### Potential sources of bias

As we used a non-obligatory neuropediatric network for patient recruitment, we acknowledge that registration of SWS cases may be incomplete. Yet, estimation of completeness is difficult as no obligatory reporting system exists in the German-speaking countries. Notably, we cannot rule out an increased inclusion of more severely affected SWS patients for this cohort as severely affected patients tend to seek medical attention in tertiary centres and university hospitals more often than mildly affected patients [52]—where rare disease networks such as ESNEK are more well-known. A differential recruiting completeness-depending on the disease severity—could result in *selection bias.* Vice versa, less severely affected patients—may have been missed by the reporting system. Potential *selection bias* would also explain why no patients with SWS Type II (no neurologic involvement) were reported in this cohort.

Using capture-recapture methods, the experience from other German pediatric rare-disease networks such as ESPED (transl.: German Pediatric Surveillance Unit) showed that overall completeness of registration ranged between 37 and 44% for Kawasaki Disease [53]. Such methods are not available for this study, as no other independent estimates for SWS prevalence are available for the German-speaking countries. For future research, an analysis of hospital records could potentially provide a remedy here.

As Type III patients display no externally visible signs of the condition, this type is especially prone to *detection bias*. Undiagnosed patients with Type III SWS, e.g. cases misclassified as migraine [4, 41] or as meningitis [47] (s.a.), escape every reporting system, resulting in underestimation of SWS Type III prevalence. Additionally, we cannot rule out underreporting of mildly affected phenotypes which may lead to overestimation of disease severity in SWS Type III cases.

### Strengths and limitations of our study

Our multinational cohort gives insight into detailed clinical profiles not only in classic SWS Type I, but also in SWS Type III, which has so far been mostly described in case reports. We included data on SWS neuroscores, use of aspirin, ASM and epilepsy surgery. As main limitations, we acknowledge that incompleteness of registration through a non-obligatory, though well-established neuropediatric network may lead to potential bias. Given the rarity of SWS, the small sample size of Type III patients may increase the probability of chance findings and it may decrease the power for the detection of significant findings. The achieved sample size restricted our possibilities for multivariable modelling. Yet, our exploratory analyses serve as a valuable basis for new directions in future research.

## Conclusions

Our findings are compatible with an incomplete, sometimes milder phenotype in Type III, FPB negative patients as compared to classic SWS Type I. The hallmarks of this to date poorly investigated SWS type are a later first diagnosis, an on average better neurocognitive development despite otherwise overall comparable epilepsy characteristics as compared to classic Type I SWS. Increased awareness and counselling on typical symptoms and imaging characteristics are necessary for more timely diagnoses in SWS Type III.

## Supplementary Information

Additional file1 (DOCX 119 kb): Figure 1, shows boxplots with median number of required antiseizure medication in surgical and non-surgical patients with SWS. 

## Data Availability

The datasets generated and/or analysed during the current study are not publicly available due concerns for patient confidentiality but are available from the corresponding author on reasonable request.

## Appendix

See Table 5.

**Table 5:** Detailed clinical characteristics of six patients with SWS included into this study which we classified as “atypical” due to *additional* features as shown in column 8

Patient number	Leptomeningeal CM	Facial CM: number of affected segments *	Age (years)	Sex†	Seizures	Glaucoma	Criterion for classification as "atypical"
1	Yes	7	4	f	Yes	Congenital	Diverse additional congenital malformations: 1. Cardiologic: Tetralogy of Fallot, diverse intrathoracic arterial malformations including retrograde perfusion of the left subclavian artery via the left vertebral artery, an absent connection of the left subclavian artery to the aortic arch, a large MAPCA (major aortopulmonary Collateral Artery) originating from the left subclavian artery to the left pulmonary artery with a high-grade stenosis and a small, tortuous MAPCA originating form the left vertebral artery to the left lung → *these malformations fulfill the criteria for "possible PHACE(S) Syndrome"* as described by Haggstrom et al., Pediatrics, 2010 *(but this would not consider the facial birthmark, ipsilateral leptomeningeal CM and congenital glaucoma, i.e. the hallmarks of SWS)* 2. Other: low-set left ear with microtia This unique case was presented at Annual Meeting of the German Society for Neonatology and Pediatric Intensive Care Medicine, Bonn, Germany in 2014 by author SD
2	Yes	7	3	m	Yes	Congenital	Classified as "systemic angiomatosis" and shows signs of Klippel-Trénaunay spectrum: 1. Extensive CM on the skin of right body half, beyond the face: occiput, back (crossing the midline), buttocks, and right upper and lower extremities → classified as signs of systemic angiomatosis 2. Signs of Klippel-Trénaunay spectrum, e.g. leg length discrepancy right > left
3	Yes	8	2	f	No	Congenital	Diverse additional malformations: 1. Synostosis of the metopic suture 2. Macrocephaly without hydrocephalus 3. Hypoplasia of the right vertebral artery and sinus transversus links (variants?)
4	Yes	10	0	m	Yes	Congenital	Signs of Klippel-Trénaunay spectrum: Entire upper body, upper extremities edematous but lower extremities hypoplastic CM on skin extends over the whole face, arms and upper body
5	Yes	11	1	m	Yes	Congenital	Classified as “overlap phacomatosis” due to additional signs of Klippel-Trénaunay spectrum: Whole right side of the body CM on the skin Hemihypertrophy of the right side of the body, especially right lower extremity
6	Yes	9	1	m	Yes	In further course	Classified as "systemic angiomatosis" Extensive, irregularly shaped CM of the skin over the whole body

*All respective facial localizations are available in our internal database but are not presented in order to maintain the clarity of the table

^†^f = female, m = male

## Abbreviations

ASM: Antiseizure medication
CI: Confidence interval
CM: Capillary malformation
ESNEK: (German “Erhebung Seltener Neurologischer Erkrankungen im Kindesalter”; English translation “Survey of Rare Neurological Disorders in Childhood”)
FPB: Facial portwine birthmark
SWS: Sturge-Weber Syndrome

## Footnotes

1 Paresis grade 3: gross and fine motricity significantly affected, grade 4: gross and fine motricity severely affected, independent gait severely compromised or not possible.

2 Powell et al.: seizure onset at 7 months [17], Day et al. 5 months/10 months for bilateral/unilateral PWS [6].

3 In some publications, the old terminology is used: simple partial or complex partial seizures.

4 The German National Guideline on Diagnostic Principles in Epilepsy in Childhood [48]—currently under revision—also does no not state routine contrast application, but recommends it in cases with “tumours,vascular malformations, inflammation or infections.

## References

[1] Roach ES. Neurocutaneous syndromes. Pediatr Clin N Am. 1992;39(4):591–620. 10.1016/s0031-3955(16)38367-5 1635798

[2] Phung T. Vascular Anomalies [cited 2025 Mar 27]. https://www.issva.org/UserFiles/file/ISSVAUpdatedflowdiagram_03152025_copyright_3.21.25.pdf.

[3] Aydin A, Cakmakçi H, Kovanlikaya A, Dirik E. Sturge-Weber syndrome without facial nevus. Pediatr Neurol. 2000;22(5):400–2. 10913734 10.1016/s0887-8994(00)00127-2

[4] Jordan PR, Iqbal M, Prasad M. Sturge-Weber syndrome type 3 manifesting as 'Status migrainosus'. BMJ Case Rep. 2016; 2016. 10.1136/bcr-2016-216842 PMC5174775 27993821

[5] Ferrari L, Coppi E, Caso F, Santangelo R, Politi LS, Martinelli V, et al. Sturge–Weber syndrome with an unusual onset in the sixth decade: a case report. Neurol Sci. 2012;33(4):949–50. 22020422 10.1007/s10072-011-0822-y

[6] Smegal LF, Sebold AJ, Hammill AM, Juhász C, Lo WD, Miles DK, et al. Multicenter research data of epilepsy management in patients with Sturge–Weber syndrome. Pediatr Neurol. 2021;119:3–10. 33813331 10.1016/j.pediatrneurol.2021.02.006 PMC8162684

[7] Jagtap S, Srinivas G, Harsha KJ, Radhakrishnan N, Radhakrishnan A et al. Sturge-Weber syndrome.

[8] Kaplan EH, Kossoff EH, Bachur CD, Gholston M, Hahn J, Widlus M et al. Anticonvulsant efficacy in Sturge-Weber syndrome. 10.1016/j.pediatrneurol.2015.10.015 PMC4943018 26997037

[9] Day AM, McCulloch CE, Hammill AM, Juhász C, Lo WD, Pinto AL et al. Physical and family history variables associated with neurological and cognitive development in Sturge-Weber syndrome. 10.1016/j.pediatrneurol.2018.12.002 PMC7288445 30853154

[10] Cho S, Maharathi B, Ball KL, Loeb JA, Pevsner J. Sturge–Weber syndrome patient registry: delayed diagnosis and poor seizure control. J Pediatr. 2019;215:158-163.e6. 31587863 10.1016/j.jpeds.2019.08.025

[11] Sánchez-Espino LF, Ivars M, Antoñanzas J, Baselga E. Sturge-Weber syndrome: a review of pathophysiology, genetics, clinical features, and current management approache. Appl Clin Genet. 2023;16:63–81. 37124240 10.2147/TACG.S363685 PMC10145477

[12] Waelchli R, Aylett SE, Robinson K, Chong WK, Martinez AE, Kinsler VA. New vascular classification of port-wine stains: improving prediction of Sturge-Weber risk. Br J Dermatol. 2014;171(4):861–7. 24976116 10.1111/bjd.13203 PMC4284033

[13] Dutkiewicz A-S, Ezzedine K, Mazereeuw-Hautier J, Lacour J-P, Barbarot S, Vabres P, et al. A prospective study of risk for Sturge-Weber syndrome in children with upper facial port-wine stain. J Am Acad Dermatol. 2015;72(3):473–80. 25592619 10.1016/j.jaad.2014.11.009

[14] Dymerska M, Kirkorian AY, Offermann EA, Lin DD, Comi AM, Cohen BA. Size of facial port-wine birthmark may predict neurological outcome in Sturge-Weber syndrome. 10.1016/j.jpeds.2017.05.053 PMC6924278 28711177

[15] El Hachem M, Diociaiuti A, Galeotti A, Grussu F, Gusson E, Ferretti A, et al. Multidisciplinary, multicenter consensus for the care of patients affected with Sturge-Weber syndrome. Orphanet J Rare Dis. 2025;20(1):28. 39819452 10.1186/s13023-024-03527-w PMC11740666

[16] Sabeti S, Ball KL, Bhattacharya SK, Bitrian E, Blieden LS, Brandt JD, et al. Consensus statement for the management and treatment of Sturge-Weber syndrome: neurology, neuroimaging, and ophthalmology recommendations. Pediatr Neurol. 2021;121:59–66. 34153815 10.1016/j.pediatrneurol.2021.04.013 PMC9107097

[17] Powell S, Fosi T, Sloneem J, Hawkins C, Richardson H, Aylett S. Neurological presentations and cognitive outcome in Sturge-Weber syndrome. Eur J Paediatr Neurol. 2021;34:21–32. 34293629 10.1016/j.ejpn.2021.07.005

[18] Shirley MD, Tang H, Gallione CJ, Baugher JD, Frelin LP, Cohen B, et al. Sturge-Weber syndrome and port-wine stains caused by somatic mutation in GNAQ. N Engl J Med. 2013;368(21):1971–9. 23656586 10.1056/NEJMoa1213507 PMC3749068

[19] Sundaram SK, Michelhaugh SK, Klinger NV, Kupsky WJ, Sood S, Chugani HT, et al. GNAQ mutation in the venous vascular malformation and underlying brain tissue in Sturge-Weber syndrome. Neuropediatrics. 2017;48(5):385–9. 28571101 10.1055/s-0037-1603515 PMC5587372

[20] Hildebrand MS, Harvey AS, Malone S, Damiano JA, Do H, Ye Z, et al. Somatic GNAQ mutation in the forme fruste of Sturge-Weber syndrome. Neurol Genet. 2018;4(3): e236. 29725622 10.1212/NXG.0000000000000236 PMC5931068

[21] Polubothu S, Al-Olabi L, Del Carmen BM, Chacko A, Eleftheriou G, Glover M, et al. GNA11 mutation as a cause of Sturge-Weber syndrome: expansion of the phenotypic spectrum of Gα/11 mosaicism and the associated clinical diagnoses. J Investig Dermatol. 2020;140(5):1110–3. 31838126 10.1016/j.jid.2019.10.019 PMC7187890

[22] Fjær R, Marciniak K, Sundnes O, Hjorthaug H, Sheng Y, Hammarström C, et al. A novel somatic mutation in GNB2 provides new insights to the pathogenesis of Sturge-Weber syndrome. Hum Mol Genet. 2021;30(21):1919–31. 34124757 10.1093/hmg/ddab144 PMC8522634

[23] Fjær R. 2021-Fjaer_new somatic mutation in GNB2 provides new insights to the pathogenesis of Sturge-Weber syndrome. 10.1093/hmg/ddab144 PMC8522634 34124757

[24] Thorpe J, Frelin LP, McCann M, Pardo CA, Cohen BA, Comi AM, et al. Identification of a mosaic activating mutation in GNA11 in atypical Sturge-Weber syndrome. J Investig Dermatol. 2021;141(3):685–8. 32771470 10.1016/j.jid.2020.03.978 PMC8483769

[25] Zhang D, Sánchez-Espino LF, Ivars M, Pope E, Nopper AJ, Arkin LM et al. Phenotypic spectrum of GNA11 R183C mosaicism. Pediatr Dermatol. 2024. 10.1111/pde.15802 PMC12118530 39654261

[26] Wu Y, Peng C, Huang L, Xu L, Ding X, Liu Y, et al. Somatic GNAQ R183Q mutation is located within the sclera and episclera in patients with Sturge-Weber syndrome. Br J Ophthalmol. 2022;106(7):1006–11. 33707187 10.1136/bjophthalmol-2020-317287 PMC9234408

[27] Valery CB, Comi AM. Sturge-Weber syndrome: updates in pathogenesis, diagnosis, and treatment. Ann Child Neurol Soc. 2023;1(3):186–201. 10.1002/cns3.20031 PMC13359178 42563829

[28] Yeom S, Cohen B, Weiss CR, Montano C, Wohler E, Sobreira N, et al. Genetic testing in the evaluation of individuals with clinical diagnosis of atypical Sturge-Weber syndrome. Am J Med Genet A. 2023;191(4):983–94. 36710374 10.1002/ajmg.a.63106

[29] Disse SC, Küpper H, Bock A, Korenke G-C, Ramantani G, Weidner B, et al. The natural history of pediatric Sturge-Weber Syndrome: a multinational cross-sectional study. Eur J Paediatr Neurol. 2025;54:200–9. 39986237 10.1016/j.ejpn.2025.02.004

[30] Kelley TM, Hatfield LA, Lin DDM, Comi AM. Quantitative analysis of cerebral cortical atrophy and correlation with clinical severity in unilateral Sturge-Weber syndrome. 10.1177/08830738050200110201 16417855

[31] Brockmann K. Erhebung Seltener Neurologischer Erkrankungen im Kindesalter. Neuropediatrics 2014; 45(S 01).

[32] Disse SC, Toelle SP, Schroeder S, Theiler M, Weibel L, Broser P, et al. Epidemiology, clinical features, and use of early supportive measures in PHACE syndrome: a European multinational observational study. Neuroepidemiology. 2020;54(5):383–91. 32610335 10.1159/000508187

[33] Kavanaugh B, Sreenivasan A, Bachur C, Papazoglou A, Comi A, Andrew Zabel T. Intellectual and adaptive functioning in Sturge-Weber syndrome. 10.1080/09297049.2015.1028349 PMC4868126 25952468

[34] RStudio. R: a language and environment for statistical computing. Version version 1.2.1335 ed2017. Vienna, Austria: R Foundation for Statistical Computing. https://www.R-project.org.

[35] Bosnyák E, Behen ME, Guy WC, Asano E, Chugani HT, Juhász C. Predictors of cognitive functions in children with Sturge-Weber syndrome: a longitudinal study. 10.1016/j.pediatrneurol.2016.05.012 PMC4983234 27353695

[36] Luat AF, Juhász C, Loeb JA, Chugani HT, Falchek SJ, Jain B, et al. Neurological complications of sturge-weber syndrome: current status and unmet needs. Pediatr Neurol. 2019;98:31–8. 31272784 10.1016/j.pediatrneurol.2019.05.013

[37] 2018-Tekin_SWS type III.

[38] Maraña Pérez AI, Ruiz-Falcó Rojas ML, Puertas Martín V, Domínguez Carral J, Carreras Sáez I, Duat Rodríguez A, et al. Analysis of Sturge-Weber syndrome: a retrospective study of multiple associated variables. Neurología (English Edition). 2017;32(6):363–70. 10.1016/j.nrl.2015.12.012 26964511

[39] Zhang Y, Niu J, Wang J, Cai A, Wang Y, Wei G, et al. Neurological function and drug-refractory epilepsy in Sturge-Weber syndrome children: a retrospective analysis. Eur J Pediatr. 2024;183(4):1881–90. 38305888 10.1007/s00431-024-05448-z

[40] Kossoff EH, Bachur CD, Quain AM, Ewen JB, Comi AM. EEG evolution in Sturge-Weber syndrome. Epilepsy Res. 2014;108(4):816–9. 24560844 10.1016/j.eplepsyres.2014.01.023 PMC4114141

[41] Huang HY, Lin K-H, Chen J-C, Hsu Y-T. Type III Sturge-Weber syndrome with migraine-like attacks associated with prolonged visual aura. Headache. 2013;53(5):845–9. 23465081 10.1111/head.12067

[42] Crosley CJ, Binet EF. Sturge-Weber Syndrome: presentation as a focal seizure disorder without nevus flammeus. Clin Pediatr (Phila). 1978;17(8):606–9. 97040 10.1177/000992287801700801

[43] Jagtap SA, Srinivas G, Radhakrishnan A, Harsha KJ. A clinician’s dilemma: Sturge-Weber syndrome ’without facial nevus’!! Ann Indian Acad Neurol. 2013;16(1):118–20. 23661980 10.4103/0972-2327.107725 PMC3644771

[44] Kim W, Kim J-S, An J-Y, Lee S-J, Chung S-R, Kim Y-I, et al. Sturge-Weber syndrome, without a facial port-wine stain, with epilepsy onset in the fifth decade. Epileptic Disord. 2008;10(1):76–7. 18484115

[45] Sen Y, Dilber E, Odemis E, Ahmetoglu A, Aynaci FM. Sturge-Weber syndrome in a 14-year-old girl without facial naevus. Eur J Pediatr. 2002;161(9):505–6. 12418457 10.1007/s00431-002-1033-6

[46] Taly AB, Nagaraja D, Das S, Shankar SK, Pratibha NG. Sturge-Weber-Dimitri disease without facial nevus. Neurology. 1987;37(6):1063–4. 3587631 10.1212/wnl.37.6.1063

[47] Muralidharan V, Failla G, Travali M, Cavallaro TL, Politi MA. Isolated leptomeningeal angiomatosis in the sixth decade of life, an adulthood variant of Sturge Weber Syndrome (Type III): role of advanced Magnetic Resonance Imaging and Digital Subtraction Angiography in diagnosis. BMC Neurol. 2020;20(1):366. 33023482 10.1186/s12883-020-01944-5 PMC7541244

[48] Trollmann R. Leitlinien 2017_BNAH_korr AH 27-11-17 [cited 2025 Mar 26]. https://register.awmf.org/assets/guidelines/022-007l_S1_Diagnostische-Prinzipien-bei-Epilepsien-des-Kindesalters_2018-03.-abgelaufen.pdf.

[49] Anand V, Vinayan KP, Radhakrishnan S. Looks can be deceiving: an appraisal of Sturge weber syndrome type III case series. Brain Dev. 2024;46(10):392. 39299834 10.1016/j.braindev.2024.09.003

[50] Hadjinicolaou A, Quinlan A, Liu S, Zhang B, Takeoka M, Sahin M, et al. Variation in neuroimaging and outcomes in patients with Sturge Weber syndrome Type III. Brain Dev. 2024;46(7):244–9. 38740533 10.1016/j.braindev.2024.05.001

[51] Ramantani G, Bulteau C, Cserpan D, Otte WM, Dorfmüller G, Cross JH, et al. Not surgical technique, but etiology, contralateral MRI, prior surgery, and side of surgery determine seizure outcome after pediatric hemispherotomy. Epilepsia. 2023;64(5):1214–24. 36869851 10.1111/epi.17574

[52] Kanafani ZA, Kanj SS, Cabell CH, Cecchi E, de Oliveira Ramos A, Lejko-Zupanc T, et al. Revisiting the effect of referral bias on the clinical spectrum of infective endocarditis in adults. Eur J Clin Microbiol Infect Dis. 2010;29(10):1203–10. 20549531 10.1007/s10096-010-0983-2

[53] Jakob A, Whelan J, Kordecki M, Berner R, Stiller B, Arnold R, et al. Kawasaki disease in germany: a prospective, population-based study adjusted for underreporting. Pediatr Infect Dis J. 2016;35(2):129–34. 26465100 10.1097/INF.0000000000000953

