# Supplementary figures and images for: Sturge Weber syndrome in a multinational pediatric cohort: a systematic analysis of different types
Source: Orphanet J Rare Dis. 2025 Jul 2;20:336. doi: 10.1186/s13023-025-03769-2 (PMC12225184; doi:10.1186/s13023-025-03769-2)

**Figure 1** (only-only, supplementary content)


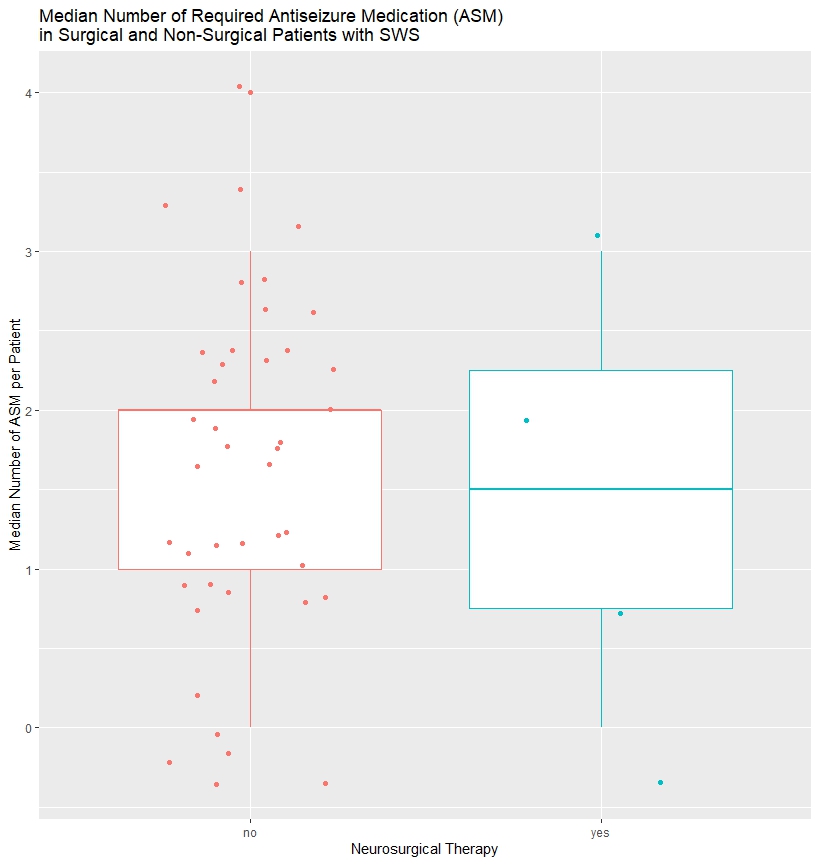

Supplement: Supplementary file 1 — Additional file1 (DOCX 119 kb): Figure 1, shows boxplots with median number of required antiseizure medication in surgical and non-surgical patients with SWS. [file 13023_2025_3769_MOESM1_ESM.docx]
